# Supplementary material for: ‘Normal’ hearing thresholds and fundamental auditory grouping processes predict difficulties with speech-in-noise perception
Source: Sci Rep. 2019 Nov 14;9:16771. doi: 10.1038/s41598-019-53353-5 (PMC6856372; doi:10.1038/s41598-019-53353-5)
Supplement: Supplementary file 1 — Supplementary Figure [file 41598_2019_53353_MOESM1_ESM.pdf]

‘Normal’ hearing thresholds and fundamental auditory grouping processes predict difficulties with speech-in-noise perception

Emma Holmes[1] and Timothy D. Griffiths[1,2]

[1]Wellcome Centre for Human Neuroimaging, UCL, London, U.K.

[2]Institute of Neuroscience, Newcastle University, Newcastle upon Tyne, U.K.

Corresponding author:

Corresponding author: Emma Holmes; E-mail: emma.holmes@ucl.ac.uk; Phone: +44 7597 967397; Mailing address: Wellcome Centre for Human Neuroimaging, UCL Queen Square Institute of Neurology, University College London, 12 Queen Square, London WC1N 3BG, U.K.

Same-frequency figure-ground discrimination

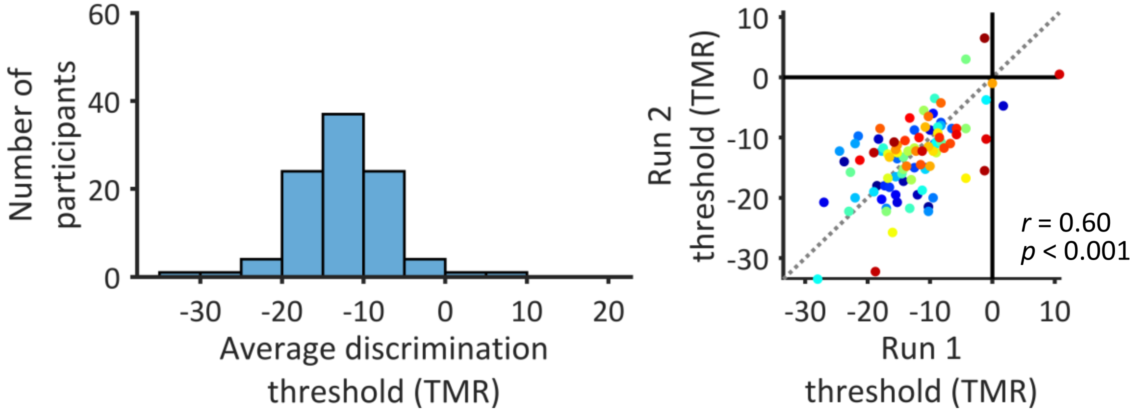

Coherent roving figure-ground discrimination

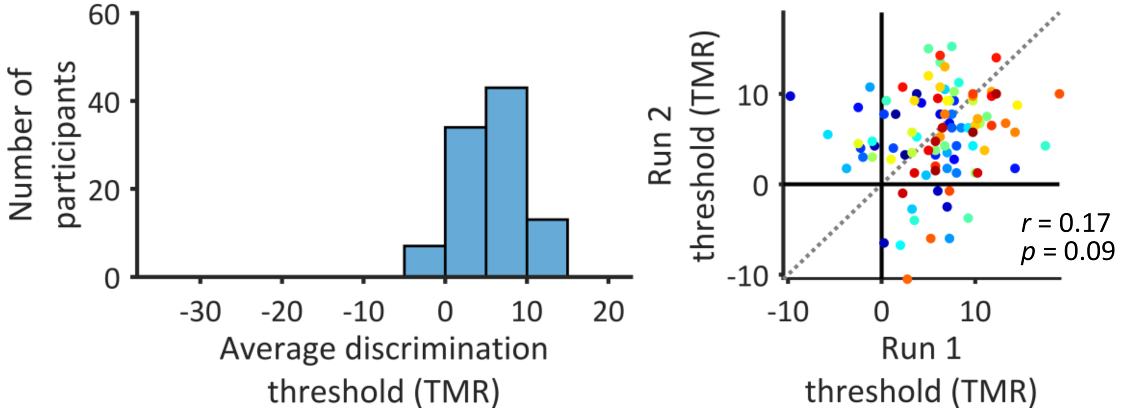

Complex roving figure-ground discrimination

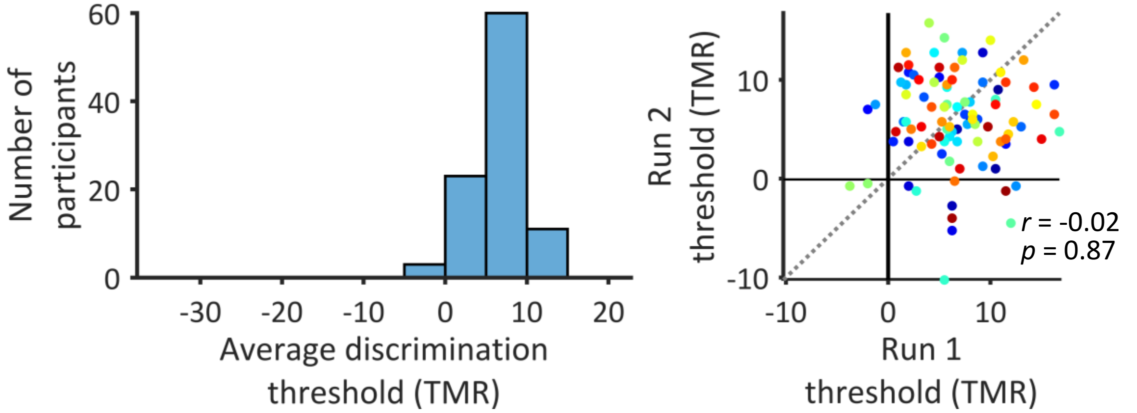

Supplementary Figure. Left panel: Histograms displaying the distribution of discrimination thresholds across participants. For each participant, the threshold is averaged across the two runs. Right panel: Scatterplots displaying the thresholds in the two (interleaved) runs of each task. Each dot displays the results of an individual participant, with the same colour coding as Figure 2B (i.e., coloured according to speech-in-noise performance). Dashed diagonal lines indicate x=y. Displayed on each figure are the coefficients and p-values associated with a Pearson's correlation across participants
